# Selling a service: experiences of peer supporters while promoting exclusive infant feeding in three sites in South Africa

**DOI:** 10.1186/1746-4358-5-17

**Published:** 2010-10-26

**Authors:** Lungiswa L Nkonki, Karen L Daniels

**Affiliations:** 1 Health Systems Research Unit, Medical Research Council, PO Box 19070, Tygerberg, 7505, South Africa; 2 Centre for International Health, University of Bergen, PO Box 7804, N-5020 Bergen, Norway

## Abstract

**Background:**

Even though it has been shown that peer support to mothers at home helps to increase exclusive breastfeeding, little is known about the experiences of peer supporters themselves and what is required of them to fulfil their day-to-day tasks. Therefore, a community-based randomised control trial using trained "lay" women to support exclusive infant feeding at home was implemented in three different sites across South Africa. The aim of this paper is to describe the experiences of peer supporters who promote exclusive infant feeding.

**Methods:**

Three focus group discussions were held, in a language of their choice, with peer supporters. These meetings focused on how the peer educators utilised their time in the process of delivering the intervention. Data from the discussions were transcribed, with both verbatim and translated transcripts being used in the analysis.

**Results:**

Unlike the services provided by mainstream health care, peer supporters had to market their services. They had to negotiate entry into the mother's home and then her life. Furthermore, they had to demonstrate competence and come across as professional and trustworthy. An HIV-positive mother's fear of being stigmatised posed an added burden - subsequent disclosure of her positive status would lead to an increased workload and emotional distress. Peer supporters spent most of their time in the field and had to learn the skill of self-management. Their support-base was enhanced when supervision focused on their working conditions as well as the delivery of their tasks. Despite this, they faced other insurmountable issues, such as mothers being compelled to offer their infants mixed feeding simultaneously due to normative practices and working in the fields postpartum.

**Conclusion:**

Designers of peer support interventions should consider the skills required for delivering health messages and the skills required for selling a service. Supportive supervision should be responsive both to the health care task and the challenges faced in the process of delivering it.

**Trial registration:**

NCT00297150.

## Background

Between 1990 and 2004 South Africa was amongst the worst performing countries in terms of its attempts to reach the child survival Millennium Development Goal (MDG) [1]. Since then under 5 mortality has increased from 67 per 1000 live births in 2004 to 72 per1000 live births in 2006 [2]. In other words, South Africa has gradually been moving away from the MDG for child survival.

Exclusive Breastfeeding (EBF) has been identified as one of the key interventions for achieving the MDG for child survival [1]. However, in high HIV prevalence settings breastfeeding carries some risk of transmission [3]. Formula feeding eliminates the risk of transmission, but increases the risk of diarrhoea and pneumonia in resource-poor settings [4]. In trying to eliminate mother-to-child transmission of HIV, the World Health Organization (WHO) recommended avoidance of all breastfeeding by HIV-infected mothers in contexts where replacement feeding is acceptable, feasible, affordable, sustainable and safe [5]. If all these conditions are not met, exclusive breastfeeding for the first few months should be initiated and stopped as soon as the desired conditions have been met [5]. However, findings from a local cohort study suggested that even in the context of freely available formula, mothers feed their babies breast and formula milk simultaneously (mixed feeding) [6]. This kind of feeding is considered harmful to child health, especially in settings where HIV prevalence is high. Several studies have documented an increased risk of HIV transmission in the context of mixed feeding as opposed to exclusive breastfeeding [7-9]. Furthermore, qualitative studies have highlighted difficulties when trying to maintain exclusive feeding methods during the postnatal period, pointing to an urgent need for infant feeding support beyond the antenatal stage [10,11].

This need was not unique to South Africa - other low and middle income countries were facing the same challenge. However, the difference between South Africa and these other countries was the significantly higher HIV prevalence in South Africa. Therefore, when interventions to promote infant feeding beyond the antenatal period were designed, the promotion of exclusive breastfeeding was the main focus. Randomised controlled trials from Brazil, Philippines, Mexico and Bangladesh have shown that a community-based peer support intervention can improve breastfeeding rates substantially [12-16]. However, none of these success stories hailed from an African setting. These interventions varied in the timing and follow-up period of visits, counselling material and training of peer supporters. It was only recently that a study from an African setting has been published [17]. Bland et al demonstrated that it is possible to promote and sustain exclusive breastfeeding for six months in both HIV-positive and HIV-negative women through home support using lay health workers [1].

It was against the latter background that an intervention (PROMISE EBF) was designed to improve the rates of exclusive infant feeding through the assistance of community peer supporters. PROMISE EBF was implemented in three study sites amongst HIV-positive and HIV-negative women. These sites represented the variety of settings that exist in South Africa in three respects, namely: area of residence, antenatal HIV prevalence and health systems functioning [18]. The chosen sites included a peri-urban farm, a rural village and an urban township. The antenatal HIV prevalence was 12.6%, 26.0% and 37.4%, respectively [19]. The PROMISE EBF has been evaluated through several research approaches including a cluster randomised controlled trial, an economic evaluation and several small qualitative studies. We report on a qualitative study that was part of the economic evaluation. The aim of this paper is to describe the experiences of peer supporters promoting exclusive infant feeding (EBF or Exclusive Formula Feeding [EFF]) in South Africa.

### The intervention

PROMISE EBF was a multi-centre community randomised trial conducted in four sub-Saharan African countries, namely Burkina Faso, South Africa, Uganda and Zambia.

During the course of routine antenatal care and hospital deliveries, expectant mothers were (ideally) offered voluntary counselling and testing for HIV and counselled on infant feeding choices. At this point the mothers are expected to be in a position to choose between EBF and EFF. The role of the peer supporter followed from this stage of routine care. One of the first tasks was to establish what feeding choice the mother had made in order to continue supporting the mother in her choice. Most importantly, the peer supporter had to discourage mixed feeding and effectively explain the dangers associated with it. Peer supporters conducted home visits to support infant feeding and promote child health at least once at the antenatal stage and at one week, four weeks, seven weeks and 10 weeks post-delivery, with a possibility of an extra visit, when necessary.

Since this was a randomised controlled trial, there were two groups of peer supporters, divided between intervention and control groups. The intervention group had peer supporters promoting infant feeding while the control group mainly assisted mothers with accessing child grants and other social services.

Peer supporters for the intervention were selected on the criteria that they had completed 12 years of schooling, had an interest in child health, had prior experience of community involvement and resided within the selected trial clusters. There was neither an age limit nor a requirement for them to have personally breastfed. Peer supporters had to successfully complete literacy and basic counselling skills assessment tests. They received five training sessions, the first being a WHO/United Nations Children's Fund (UNICEF) HIV and Infant Feeding Counselling Course [20]. The subsequent four training sessions were developed in response to their needs as identified by peer supporters as the intervention progressed. These one- or two-day sessions included: HIV (disclosure and transmission), computer training and workshops on care-giving and discipline. Peer supporters were initially paid a monthly stipend of R1000 (US$127, *to convert to US Dollars we used the average annual exchange rate for 2007, 1 US Dollar = R7.9*[6]), in the second year of the trial the stipend increased to R1200 (US$152). Peer supporters commenced work in September 2005 and had completed follow-up work of all women recruited for peer support by December 2007.

The economic evaluation measured costing with the use of an activity-based approach. By so doing peer support was identified as an activity. Estimating the costs of peer support included calculating the time spent on various activities. Peer supporters were asked to document their activities. In addition, focus group discussions (FGD) were held with peer supporters to recall how they spent their time. The FGD was an appropriate data collection technique for three reasons. In the first instance, it validated the quantitative tool. Secondly, it identified a full range of perspectives from peer supporters on their time spent during the intervention. Thirdly, the group context afforded peer supporters an opportunity to discuss issues raised by their colleagues which would have been easily neglected in an in-depth interview. These reasons are amongst those outlined by Powell and Single for conducting focus groups [21]. The discussions in focus groups were richer than anticipated with participants not only describing how they spent their time but also offering valuable insights into the broader context of being a peer supporter. We felt that it was important to analyse and report on these FGDs and not just use them as data in support of the economic evaluation. The results of the focus group analysis are therefore also presented in this paper.

## Methods

During June 2007 one FGD was conducted by the first author in each of the three intervention sites. The FGDs where held immediately after the peer supporters meeting at the study offices where they usually had their monthly meetings. Having the FGDs coinciding with the monthly meeting was convenient for peer supporters - the location was central to all of them. The interviews were conducted in the peer supporters' first language, isiXhosa and isiZulu in two sites; the third site preferred Afrikaans, isiXhosa and English. It was requested that questions remain in English with the response being delivered in either isiXhosa or Afrikaans. The FGD questions are listed in Table 1.

**Table 1:** Questions explored during the FGD

Theme	Questions
**Recruitment**	Could you please explain how you recruited women to the study?
	How long did it take you to walk around looking for women to recruit?
	How long did it take you to recruit women when you had scheduled visits that you needed to honour?

**Visits**	Could you please explain what you discussed in each visit?
	a. How long was each visit?
	If you find the baby sick during any of the visits, what did you do?
	b. How long would this visit take?
	Did you conduct more than the specified reasons, in other words did you have extra visits?
	c. How many extra visits did you do per person?

**Disclosure**	Did the women you visited disclose to you?
	d. When did most women disclose?

**Planning**	How did you decide on the number of visits you needed to do per day?
	Did you plan at the beginning of the week for the whole week or did you plan daily just for that particular day? How did you manage this planning?
	e. How long did the planning take?

**Breaks during work**	When you do this work do you take lunch?
	f. If yes how long is your lunch?
	g. If no, why not?

**Supervision**	Did you have monthly meetings?
	h. If yes, what did you discuss?
	i. How long were these meetings?
	Did you have one on one meeting with your supervisor? Were you ever accompanied by a supervisor to a visit?
	j. Were these visits longer or shorter?
	k. After a visit with your supervisor did you discuss your work afterwards?

**Missed visits**	Did you ever miss a visit?
	l. What are the reasons for missing a visit?

**Travel**	Did you ever use public transport in your work?
	m. If yes, how much did it cost?

	Is there anything else you would like to talk about?

Thank you all for participating in this focus group discussion.	

The FGDs lasted between 60-80 minutes. The discussions were audio recorded, transcribed verbatim and then translated into English by an independent transcriber and translator. To verify the accuracy of the transcription and translation, the first author read the transcripts checking them against the audio recorded interviews. Seven peer supporters participated in each of the urban and rural focus groups, while five participated in the peri-urban ones. All peer supporters working in the intervention arm agreed to participate.

Each peer supporter's main responsibility was to recruit pregnant women into the intervention group and thereafter conduct at least five follow-up visits. In the FGD peer supporters were asked to describe each task they were required to do and the amount of time they spent on that task. For example, they were asked to describe how they had recruited women to participate in the intervention.

Written informed consent was obtained and all participants were informed that they could refuse participation or withdraw from the discussions at any time. The University of the Western Cape granted the necessary ethical approval to conduct the intervention.

The first author had initially been employed as an assistant project manager on this intervention and was involved in the selection of the peer supporters who were employed in the intervention. Prior to conducting the FGDs she was concerned that the peer supporters would not share information openly with her if they perceived her as being involved in managing the intervention. However, during the course of the FGDs this concern was abated since peer supporters shared information openly and especially voiced their discomforts. Upon reading the transcripts the second author, who did not form part of the intervention team, confirmed that the peer supporters were more than willing to disclose information freely during these discussions.

### Analysis

A thematic analysis of the data based on data immersion was conducted [22]. Each transcript was read by the authors individually. Lungiswa Nkonki (LN) worked with the transcripts in their original language, while Karen Daniels (KD) read the translated versions. Each transcript was analysed independently. After immersing herself in each transcript, LN graphically depicted the story each transcript told. She was then able to develop key words describing the data. Such key words were grouped together into meaningful patterns and thereafter created themes representing a more conceptual understanding. LN returned to the transcripts to validate each theme with quotes and marked quotes that corresponded with each theme. Quotes were printed and grouped on flip chart-sized sheets.

Both authors read the themes with their authenticating quotes and clarified interpretations. The authors reached consensus on the commonality of the themes between the sites and agreed on combining the themes. In some instances, the peer supporters' experiences across the three sites revealed certain particulars in emphasis of their own experiences in a peri-urban area, rural area and urban township.

**Figure 1:**
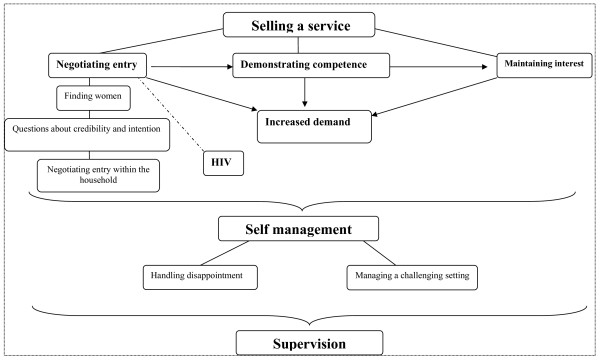
**A map of the process of delivery the intervention from peer supporters' perspective**.

Three main themes emerged: selling a service, self management and supervision. Using these themes LN constructed a model explaining the data across all three interviews (see Figure 1). The results presented below explain the data against the background of this model. The results are a summary of the analysis process with selected quotes included for illustration purposes. These results were validated through a presentation to the broader research team and later through a presentation at a local public health conference.

## Results

The model explains peer supporters' experiences in delivering the message of exclusive infant feeding to women (Figure 1). We have tried to give voice to all the facets of peer supporter experiences in this program. Our findings highlight that delivering the peer support intervention mainly involved selling a service. This meant peer supporters had to convince both the pregnant woman and her household about the value and potential usefulness of receiving peer support. However, gaining access to the woman (mother) was not enough. Peer supporters had to demonstrate that they indeed had more knowledge on infant feeding and child health, in order to retain the mother's need for remaining visits. Once a peer supporter had undergone this process with the mothers, they also received requests for visits from other women who were not even enrolled in the study. These women were not only interested in infant feeding, but also had questions on maternal health, family problems and access to social services.

HIV added a complexity to peer supporters' efforts to deliver the intervention. The recruitment of women into the study was not based on their HIV status. Even though this was the case, women associated private visits with HIV and did not want to be associated with HIV programmes. Subsequently, in negotiating entry peer supporters had to clearly disassociated the program from HIV. But when the intervention started peer supporters were encouraged to elicit the women's status. Knowing a woman's status would help the peer supporter provide a more tailored message and therefore enhance her work. In practice peer supporters found disclosures to be time consuming and distressing.

In delivering this intervention peer supporters largely had to self-manage. This included managing their time and challenging situations in the field. Despite their independence, the peer supporters were also supervised. Even though the framework for supervision was the same, supervision in each site took a different format.

We explain the above findings in detail below.

### Selling a service

In the current health system patients access healthcare as they feel they need it, by presenting themselves at healthcare centres. This can be in instances of ill health or when the antenatal care is required. Peer supporters in this study were however working backwards, given the fact that they offered mothers a service which they themselves had not expressed a need for. The data suggests that peer supporters had to sell a service to pregnant women and the community at large. Selling this service encompassed five categories: negotiating entry; negotiating competence; maintaining interest; increasing demand and stress connected to HIV disclosure; and role confusion.

### Negotiating entry

#### Finding pregnant women

Peer supporters were asked to recruit 10 mothers per month. This number was mainly influenced by the trial context. Recruiting 10 mothers per month would ensure adequate numbers for data collection. The peer supporters' first responsibility was to identify pregnant women to whom they could deliver the intervention. Initially, they used a combination of recruitment methods, including approaching women on the streets, and door-to-door recruiting. Recruiting women from the antenatal clinic was preferred by peer supporters who lived close to the clinic. As the study progressed the study team was concerned that this method would exclude women who were not attending the clinics. Therefore, peer supporters were asked to recruit directly from their communities. Community recruitment, however, was described as laborious:

*"What made it a bit difficult to get them at times was because we were told not to work from the clinics, and we would then go to their homes. Finding them at the clinics was better for us, than having to hunt for them *[in the community]*."*

At the clinic peer supporters had a pool of pregnant women ready to recruit from; in the community they would visit many houses without necessarily finding many pregnant women. This was problematic because their performance was measured against the number of women they were able to recruit.

#### Questions about credibility and intention

Even after pregnant women were identified the process of getting them to agree to participate in the intervention was challenging. Peer supporters described being cross-questioned by mothers wanting to know how this intervention would benefit them as peer supporters. For example, some respondents recalled mothers asking whether or not they were being paid to visit them. In particular, mothers were concerned that peer supporters were using these visits as means to gaining a personal income and they did not want to be used in what they suspected was the peer supporters' process of enriching themselves.

"You would visit a mother at her home and recruit her, and her reaction would be whether you would be paid for interviewing her. She would tell you that she did not want to be involved in your business as much as she did not want to sustain your life."

For the peer supporters this questioning by the mothers suggested a lack of trust. They found that they managed to allay mothers' fears only through delivering careful explanations that they were simply there for the good of the mothers - thoroughly explaining the nature of the support being offered. Furthermore, they asked the antenatal clinic staff if they could promote the intervention by speaking to pregnant women in the waiting rooms at the clinics. They felt that if the women first heard about the intervention at the clinic they would be less suspicious of the credibility of peer supporters.

Apart from dealing with the mothers' concerns about the purpose of their visits, peer supporters also had to field suspicion from the mothers' male partners. Lengthy visits were required when peer supporters had to clarify that their visits were only about infant feeding. According to the peer supporters male partners often thought mothers were discussing their relationships and complaining about their (partners') behaviour. In instances where the male partner disapproved of peer support, the visit would not even last five minutes.

#### Negotiating entry within the household

Once the peer supporters had recruited the mother, they felt that they needed to understand who the dominant household members were and what their influence over the mother was. In the peri-urban and urban township dominant household members included the husband or male partner as well as female relatives (aunt, sister or mother). Due to the openness of such household members, peer support work was essential and could facilitate access to the pregnant mother. In the rural site the infant's grandmother was more dominant. The paternal grandmother was particularly dominant but the maternal grandmother also had a strong influence over the mother. In these settings paternal grandmothers had to give their consent for their daughters-in-law to receive peer support.

". . . I would have to approach an elder in that home and explain that I visit expectant mothers at their homes to help them raise their babies in the proper way of breastfeeding. It is usually a mother-in-law that I have to speak to about her pregnant daughter-in-law. Having been granted permission, the mother-in-law allows me to talk to the pregnant mother."

Another important component of negotiating entry within the household was deciding on a convenient visiting time for the mother. In the urban site the late morning was more acceptable whereas in the rural village arriving early was the preferred option given the long walking distances between households. However, in the rural site early mornings were not always appropriate as newly-weds have early-morning chores which they have to do. Such chores involve fetching fire wood, cleaning and preparing breakfast; if the mother had gone to fetch fire wood the peer supporters opted to wait for them to return in order to avoid repeating the trip the next day. This often led to unproductive time.

". . . To avoid coming back the following day, I have to wait for the ones I could not find in order to finish on the same day. Our villages have transport problems and one has to walk distances on foot, which is why it is better to wait and finish everyone rather than to come again the next day. I wait for long hours at times for the ones gone to the forest to fetch fire wood till they come back at the latest hour."

HIV was a barrier to entry at household level. Pregnant women feared being visited by a peer supporter because they thought other community members might think that they are HIV-positive.

This section has highlighted that recruitment was not only a once-off activity of obtaining the woman's consent, but that peer supporters had to continuously persuade the mother and her household of the value of peer support.

### Negotiating competence

Peer supporters adapted the way of presenting themselves to the context or circumstances of each visit and implied that the extent to which the mother trusted them was linked to this presentation. HIV-positive mothers were often anxious to find an appropriate feeding method, given their status. These mothers would apprehensively articulate their breastfeeding intentions. Peer supporters addressed these situations by being calm.

". . . she would also ask you a lot of questions . . . and how to feed the baby if you are HIV-positive. The mother would worry and say all she needs is to breastfeed her baby. By now you should play it cool and give her proper answers in a calm manner."

In some instances mothers would initiate breastfeeding and later on change to feeding formula milk. In these cases peer supporters said it was necessary for them to respond patiently.

"At times you find that, while you had initially agreed on breastfeeding during the antenatal visits, the mother is now feeding the baby formula milk. With patience you then have to start motivating the mother to breastfeed and show her how to sterilize and keep the bottle clean if she has to use it."

Peer supporters had to demonstrate sound knowledge of their subject matter (infant feeding). Thus they would conduct demonstrations such as cleaning the feeding utensils for mothers who are feeding infant formula and assisting breastfeeding mothers with positioning of the infant. Furthermore, these demonstrations included observing the infant for any possible signs of illness.

Peer supporters' behaviour during visits, that is, the application of both empathetic qualities as well as demonstrating sound knowledge of infant feeding proved important in gaining the mothers' trust.

### Maintaining interest - "INTSEBENZISWANO"

Each peer supporter visit was planned to cover predefined infant feeding topics, but primarily aimed at addressing the needs of the mother. At times mothers or other household members' inquiries resulted in peer supporters having to introduce topics intended for the proceeding weeks. Peer supporters were careful not to cover too much information should the mother decide that she does not need more visits. In order to keep mothers interested for the next visit, a limited amount of information was given at every follow-up visit.

". . . We don't explain the whole thing because we want them to be eager to want to know more on the next visit."

Once peer supporters had managed to build good relationships with the mothers; the mothers then wanted to spend more time with them. In addition, mothers recommended peer supporters to other mothers and community members. This increased their overall popularity and acceptance within the communities.

"They do come, even my neighbour because they are not in the study area, they are not in my cluster, so they come to me and Sis Z [LN - the participant uses a hand gesture symbolising mothers coming to ask for help. Sis Z is a peer supporter] - they come and ask advice, those receiving grant support, they ask for advice, I give them, if they come to me."

". . . Later you discover that your job is made easier by being recognised as soon as you appear in some places. The mothers show the others that you are the person who helps pregnant mothers and babies."

### Stress connected to HIV disclosure and role confusion

The study team encouraged peer supporters to elicit HIV status of the women. The rationale for this was that if peer supporters knew the mothers' status they could provide more appropriate support on infant feeding choices. However, peer supporters' experiences of disclosure were contrary to this logic. Peer supporters did not describe disclosures as something they were eager to elicit and which, in turn, would make their jobs easier. Instead, they experienced that disclosures led to increased workloads. HIV-positive mothers often asked for information about their own social and health problems. Peer supporters saw their role as solely one of baby care and not inclusive of the mother's health.

At a personal level, peer supporters felt vicarious hurt when the mothers disclosed their HIV status. Despite the practical benefit of mothers disclosing, peer supporters were greatly distressed by news that a mother was HIV-positive. Some of them had not developed the professional boundary that would protect them from being personally affected by the news.

". . . I was much stressed last week, very stressed. When I came home I cried because I didn't expect it to happen to a younger child like that - and she was at ease, she told me, without me even asking her, she just told me that 'I am like this and I accepted it' - instead of being happy, I was devastated - I said this child doesn't know the real thing, you see? I took it personally . . ."

### Self management

Peer supporters only met their supervisors during monthly meetings and occasionally during supervisory field visits. For the rest of the month they had to manage their own time. This required organisational and time management skills. Peer supporters dedicated a lot of time to planning. Their tasks included data collection; they each had several forms aimed at making their work easier and capturing vital information about tasks completed at each visit. The first form carried detailed directions to the mothers' home. The second form captured the type and length of each visit, household members present, and topics covered during the visit. When asked how much time they spent on planning, peer supporters equated planning to recruitment.

"Planning takes up as much time as recruiting because you write down notes. You know the person's name, where she stays, her contact details, when it is convenient to contact her, how far she is with the pregnancy and when to pay her a visit. Your first planning is on the day you first meet the mother."

The demands of arranging and conducting successful home visits were evident in their weekly and daily plans. Every Sunday they would plan for the proceeding week. This involved identifying women who were due for different types of visits and scheduling a visit with them. In addition, they had to collate the different forms they (peer supporters) would have to fill in during the course of the visits. On a daily basis they had to consider the number of women to be visited on a particular day; the amount of time they planned on spending at each household; and the walking distance between households in order to avoid missing visits. Often the scheduling had to be adapted during the course of the week due to unforeseen delays and mothers not being available. These changes could mean a waste of time and transport money.

If the peer supporter arrived at a house where the mother had gone to fetch firewood they opted to wait for the mother to avoid walking the same distance again the next day. In rural areas houses are far apart and in some areas there are no proper roads. Peer supporters travelled between an hour and two between visits. Under these circumstances choosing to wait for a mother to return home was therefore justified. However, this choice meant the peer supporter would be idle for hours.

### Handling disappointment

In practice supporting women to maintain exclusive feeding was not a straightforward process. Peer supporters were faced with a myriad of issues which their training had not prepared them for. With regards to mixed feeding especially, peer supporters often felt disappointed when a mother mixed-fed her infant. They tried to remind the mothers about the importance of exclusive feeding but mothers often had better counter arguments. For instance, peer supporters said mothers would introduce other food as early as three weeks and would argue that their infants enjoy these foods. The next quote illustrates peer supporters' difficulty in convincing the mother to revert to the appropriate (and agreed) feeding method. Consequently, peer supporters' felt powerless in changing the situation and resorted to agreeing with the mother.

*". . . when you say no, where did you hear about this? The mother tells you her baby is enjoying the *[popular yoghurt brand] *and you run short of words. I then decide to warn the mother that I am not sure of what these *[popular yoghurt brand] *are made of, they only taste good. I also advise the mother to cook pumpkin and mash it for the baby rather than feed it *[popular yoghurt brand]*. They will agree on everything you tell them but still insist on feeding [*popular yoghurt brand]*. They assure me that the babies get satisfied after being fed therefore they will not stop that procedure."*

At times peer supporters were angered by the mothers' deviation from the exclusive feeding and often they felt like they were wasting their efforts. As a last resort they used the onset of infant illness as an opportunity to point out the consequences of mixed feeding.

". . . when their babies get the constipation and diarrhoea because they are mix feeding. They are also not washing the bottles. Then you tell her that you see what I have told you, I told you that the baby is going to be sick when you are doing this. Try to stick with what I have told you."

### Managing a challenging setting

The nature of a rural setting presented unique challenges. In this setting an interaction of structural factors and traditional practices negatively impacted on peer support. Running water, proper sanitation and electricity were not widely available. The main source of cooking fuel was firewood. It was therefore customary for women, particularly newly-wedded ones to fetch water, firewood and do other household chores. Doing these activities meant that they cannot breastfeed at the same time. Therefore, women started mixed feeding so that other household members could help with caring and feeding for the infant. If a peer supporter is confronted by this situation, the length of the visit is often extended.

Recruitment was also affected by the long distances, as those who were recruited at the clinic often opted to take public transport. Some areas were deserted and the peer supporters often had to ask someone to accompany them fearing they could be attacked or assaulted.

In addition to fetching firewood and doing other household chores it was also customary for first-time mothers to return to their parents' home for about three months. This often led to missed visits.

Peer supporters had to also promote healthcare utilisation through encouraging mothers to take their infants to the hospital when they fall ill. However, some women insisted on sending their children to a traditional healer. One peer supporter related how she struggled and failed to convince a mother to go to the clinic when she had a sick child. The mother claimed that she had been to the clinic with her child but was unhappy with the manner in which the health worker questioned her about the illness of her child.

"She says at the clinic they make comments like how could a small baby like this get so sick? As if it's a wonder . . ."

A peer supporter went on to explain that the mother believed that the course of her child's illness was Ibala (peer supporters described this as a dark mark on the child's back). Medically, this dark spot is not an illness, but in this context mothers believed that their infants would die if they are not sent to a traditional healer when they have this. The mother then insists on sending the child to her traditional healer in spite of the peer supporter's recommendation of returning to the clinic.

"The mother ultimately defeats you into agreeing that it is okay to do as she pleases as she refuses to go back to the clinic. That means she can go to the traditional healer.

Each peer supporter had a mobile phone. The research team provided them with airtime to ease communication with mothers. However, mobile telephone coverage is erratic in this setting. As a result planning was disrupted since peer supporters were unable to confirm appointments. In these situations missed visits were inevitable. To avoid these disruptions peer supporters made sure that they knew when mothers were expected to be away from home, such as for clinic visits or to collect social support grants.

Peer supporters' role extended beyond child health. They had to respond to other community needs. In this community where unemployment was rife, peer supporters said the biggest problem was hunger and mothers who did not have official identity documents. Social support grants were an important source of income. The child support grant is means tested and provides a cash benefit to the poor for children up to the age of six years. One of the documents required when accessing this grant is an identity document. According to the peer supporters most mothers they visited did not have the documentation and could not access social support.

"Jobs are scarce and without the ID the children's grant is not possible to get even if the mother has applied for them. The father in most instances is also unemployed and starvation kicks in. Just by glancing around that home you can notice hunger and sadness."

### Supervision

Even though peer supporters relied on self-management in the field, they were supervised and functioned within a larger research group. Peer supporters received two forms of supervision, one monthly meeting where they would meet as a group with the supervisor to report back on their experiences. Secondly, a peer supporter supervisor would accompany a peer supporter to a visit and observe her. Over and above peer supporter supervisors were supposed to visit a random sample of mothers in the absence of the peer supporters. Individual supervision occurred only in two sites. Peer supporters experienced individual supervision during a visit as an anxiety provoking experience. In one site the supervisor conducted random visits and would report back to the peer supporters.

Failure to recruit the stipulated number of mothers often evoked feelings of fear. Peer supporters worried about being viewed as ineffectual.

In two sites supervisors were seen as a resource that the peer supporters could get support from in difficult situations. The supervisors were said to encourage teamwork and to provide resources that would simplified the work, for instance, diaries. Peer supporters from the third site by contrast felt that they were not listened to when they voiced concerns about working conditions such as the use of public transport and personal safety in crime-ridden areas.

*"Maybe one of us *[peer supporter] *raises a point of abuse during the visits, she is not taken seriously and people just leave without attending to the problem. She asked another question and she was ignored again, so that upset her. We as her team had to take over and make her feel better and not neglected. Sometimes they are not interested in talking about other things with us."*

## Discussion

Findings from this study highlighted unique challenges in an intervention geared towards prevention and health promotion. This data has shown that to support exclusive infant feeding at the community level required peer counsellors who were able to sell their service. This meant they had to negotiate entry into the mother's home and into her life. Peer supporters had to continuously persuade the mothers about the value of peer support. They used demonstrating competencies as means of gaining the mothers' trust. Successful home visits required planning and time management. Peer supporters also had to learn the skill of self-management. Supervision that encompassed both a focus on their working conditions and on the delivery of their task was considered necessary. In spite of such support, the peer counsellors faced immense challenges in promoting exclusive feeding and often found that mothers resorted to mixed feeding despite initially agreeing to an exclusive feeding method.

Peer supporters are one of the many types of Community Health Workers (CHW) found in the world. Evidence from earlier CHW programmes revealed that CHWs with curatives skills received greater respect compared with those without curative skills [23]. In some instances preventive and promotion services were only well received after CHWs had built relationships of trust through delivering effective curative services [24]. Even though peer support excluded curative services, peer supporters managed to increase the demand for peer support. However, this demand was not only for child health it included maternal healthcare and broader social service support. An earlier review of national experiences in the use of CHWs found that community needs did not only include healthcare but also the provision of food and water [24]. More recently, experiences of CHWs have shown that the demands placed on them were beyond health-related work [25]. Reflecting on the experiences of this study, the demand for broader social service support highlights the importance of integration. It was useful that peer supporter supervisors had received training on accessing social services. As a consequence peer supporters could rely on them for support when confronted with demands extending beyond infant feeding. In fact, for most women, infant feeding was not the most pressing concern.

The interaction of poor living conditions, strong customary and normative feeding practices together created difficulties in changing infant feeding practices in this setting. The disempowerment demonstrated by the peer supporters' tendency to agree with a mother's decision to mixed feeding when they had failed to convince her to do so otherwise, is problematic. Their training had not adequately prepared them for these challenges. Another skill peer supporters lacked was the absence of a protective professional boundary from emotionally distressing situations. Dennis provides a lengthy list of potential adverse outcomes from peer support [26]. These findings provide evidence for at least two of those, namely: emotional over-involvement resulting in contagion stress and reinforcement of poor behaviours.

Ofosu-Amaah argues that threats to the CHW programmes include inadequate roots in the community and inadequate support from the health system [24]. The former has more resonance with our findings. In this study the community was not involved in the selection of peer supporters nor was it part of the development of the intervention.

In one of the sites peer supporters complained that they were only listened to when they had problems relating to the content of the work. For instance, if they had a question about a mother with engorged breasts this would be an important question that the supervisor would address. However, if they raised issues about their personal safety during visits this question would be ignored. Schneider et al recommended that a sustained and effective CHW presence in the South African health system would require, amongst other things, improved working conditions; and basic entitlements like leave and complaint mechanisms [27]. Data from this study strengthens this recommendation.

There is now evidence that it is feasible to promote and sustain exclusive breastfeeding for six months in both HIV-positive and HIV-negative women [17]. The quantitative evidence on the success of promoting exclusive breastfeeding alone is insufficient, since it does not provide information on how the intervention functioned in the field. Our findings contribute a qualitative component which explained how peer supporters in practice delivered this intervention. This information is essential for planning for scale-up.

## Conclusion

Many studies look at the success and failures of CHW experiments, but few actually consider the voices of the CHWs themselves. Findings from this study offer an important but often missing perspective. Peer supporters had to create and maintain a desire in mothers to use their services. We recommend that the training of infant feeding support include the following:

• Over and above infant feeding knowledge, peer supporters should be equipped with skills required for selling a service.

• Time management and planning.

• Training on developing a professional, protective boundary for emotionally distressing situations.

We further recommend that supervision be made responsive both to the health care task and the challenges faced in the process of delivering that task.

Programme planners or policy makers should consider expanding the tasks of peer supporters to include other needed services within that community such as maternal health and accessing social services.

## Competing interests

The authors declare that they have no competing interests.

## Authors' contributions

LN designed, planned the study and collected data. Both authors analysed and wrote the manuscript. All authors read and approved the final manuscript.
